# Future-oriented chance prioritarianism: survival prospects as the distributive currency

**DOI:** 10.1007/s11019-026-10353-3

**Published:** 2026-04-20

**Authors:** Tomasz Żuradzki

**Affiliations:** Institute of Philosophy & Interdisciplinary Centre for Ethics, Jagiellonian University in Kraków, ul. Grodzka 52, 31-044 Kraków, Poland

**Keywords:** Distributive theories, Welfarism, Prioritarianism, Healthcare rationing, Healthcare prevention

## Abstract

Debates over distributive justice often agree that the worse-off deserve priority, yet disagree about when and on what basis someone counts as worse-off. Two dimensions structure these disagreements: the temporal unit (lifetime vs. time-slice assessment) and the currency (welfare, resources, capabilities, or other goods). This article develops a form of time-slice prioritarianism—future-oriented chance prioritarianism—which identifies the worse-off as those with the lowest near-term survival prospects. On this view, survival chances, rather than welfare or life-years, function as the distributive currency to which diminishing marginal weight applies. After situating the view within the landscape of lifetime and time-slice prioritarianisms, I analyze a stylized vaccine-allocation case to show how weighting survival prospects can diverge from utilitarian or standard welfarist prioritarian recommendations, and how the verdict flips when we switch the reference class (whole cohort vs. infected subpopulation). I then address two conceptual challenges: (i) clarifying the factors relevant for assigning normative weight to risk concentration, when it conflicts with aggregate outcomes; and (ii) diagnosing the partition-sensitivity of who counts as worse-off and its interaction with interpretations of probability.

In early 2021, the vast majority of European countries—at least in their official vaccination guidelines—prioritized candidates for COVID-19 vaccines with regard to their future prospects with respect to COVID-19 infection. Specifically, individuals with a higher probability of dying from COVID-19 (if infected) were typically placed higher on vaccination priority lists for this reason, which in practice meant prioritizing the elderly (Wiśniowska et al. 2022). In this context, such a prioritization strategy was intended to minimize overall mortality, as explicitly stated in some policy justifications. However, according to model-based studies, it may also have reduced the total number of years of life lost in the population, given the steep age gradient in COVID-19 mortality (Bubar et al. 2021).

A similar pattern—prioritizing those with the lowest survival prospects—appears in the case of seasonal influenza vaccination. Here too, the elderly face the highest risk of death from infection, while children play a disproportionately large role in transmitting the virus. Model-based studies have suggested that preferentially vaccinating younger individuals could reduce transmission and ultimately prevent more deaths and years of life lost in the population (Galvani et al. 2007). From a standard health-economics perspective focused on maximizing aggregate health benefits, this option would therefore appear preferable. Nevertheless, vaccination recommendations—for example, those of the U.S. Centers for Disease Control and Prevention—have historically prioritized older adults alongside other high-risk groups, reflecting their elevated risk of severe morbidity and mortality, even though this approach can diverge from transmission-minimizing strategies (Smith et al. 2006). This pattern suggests that decision-makers in preventive contexts give special weight to those with the lowest survival prospects (in the above examples, the elderly, though this need not always be the case). This contrasts with standard prioritization in therapeutic contexts, where decisions are often guided by principles such as maximizing expected life-years or lifetime QALYs in the population (Giubilini et al. 2021). The central contribution of this paper is to argue that several strands of bioethical debate—particularly those concerning preventive medicine and vaccination^1^—can be understood as implicitly presupposing a form of chance prioritarianism. On this view, improvements in survival probabilities deserve greater moral weight when they benefit those whose baseline chances of survival are lower, even when doing so does not maximize expected aggregate benefits.^2^ Survival probabilities thus function both as a measure of disadvantage and as the currency to which a prioritarian weighting function is applied. The aim of this paper is not to defend this view, but rather to use it to explain real-world prioritization patterns in prevention and to diagnose the theoretical challenges it raises. To the best of my knowledge, this approach has received little explicit discussion in the literature on prioritarianism and distributive justice.

This topic is particularly important because, although prevention has long been one of the goals of Western medicine, we are now witnessing a shift toward a paradigm in which preventive strategies—especially those focused on risk reduction—play an increasingly central role in medical practice. The targeting of diseases through the management of risk factors (often via medication) is evident in the widespread use of risk-reducing drugs, such as cholesterol-lowering agents, antihypertensives, and antidiabetic medications. Increasingly, individual risks are treated as conditions in their own right, effectively turning individuals into patients—for example, into ‘cardiovascular risk patients’—whose risk profiles require intervention. Important questions therefore arise about how, and on what grounds, treatment should be weighed against prevention in healthcare prioritization (Faust & Menzel 2011; John 2025). The present paper contributes to this debate indirectly by offering a framework that helps explain real-world prioritization patterns in preventive healthcare.

In Sect. 1 I briefly review debates over lifetime versus time‑slice prioritarianism, and in Sect. 2 I introduce the distinctive idea of future‑oriented chance prioritarianism. Section 3 uses a hypothetical vaccination scenario to illustrate how this principle operates: by comparing two cohorts with different infection fatality rates and probabilities of infection, I show that prioritising lower survival prospects can lead to different allocation decisions depending on whether we ascribe risk to entire age groups or only to those who will actually be infected, and how this may conflict with utilitarian or QALY‑maximising approaches. Section 4 examines the conceptual and normative implications of the example and draws general conclusions.

## Prioritarianism, lifetime, and the future

Derek Parfit’s seminal presentation of prioritarianism states, “Benefiting people matters more the worse off these people are” (Parfit 1997, p. 213), and in a more recent article: “we have stronger reasons to benefit people the worse off these people are” (Parfit 2012, p. 401). While this principle has been thoroughly discussed in philosophy, healthcare ethics, and the healthcare economy, a surprising lack of consensus persists regarding the identification of ‘the worse off,’ even among those who agree on some form of prioritarian approach in healthcare contexts. Debates about the distribution of goods based on recipient characteristics refer to two key dimensions: temporal units and currencies.

This first dimension is particularly important because it concerns how different time periods are taken into account in identifying who is worse off (McKerlie 1997; Holtug 2003, p. 155). A lifetime welfare approach, which focuses on the distribution of well-being across an individual’s entire life, currently dominates philosophical and bioethical discussions of distributive theories (e.g., Ottersen 2013; Sharp and Millum 2018; Nielsen 2022). More precisely, prioritarianism, when understood as a form of welfarism, holds that each individual’s contribution to the moral goodness of a state of affairs is an increasing, strictly concave function of their lifetime well-being.

An influential explication of lifetime prioritarianism, presented by Adler (2012), describes a function that takes outcomes and returns a vector of numbers (a ‘well-being vector’). Each component of this vector (a ‘well-being number’) represents a person’s lifetime well-being in a specific outcome, aggregating past and expected future well-being. A well-being number is “a single number meant to indicate the quality of the entire life span of individual *i*, from birth to death” (Adler 2019, p. 75; see also Adler 2022). An outcome *x* is assigned a well-being vector *w(x) = (w*_*1*_*(x)*,* w*_*2*_*(x)*,* …*,* w*_*n*_*(x))* where *w*_*i*_*(x)* is the well-being number representing person *i*’s well-being in outcome *x*. This standard view implicitly assumes temporal additivity and atomism, treating lifetime well-being as the sum of well-being across sub-lifetime periods (Ponthiere 2016, p. 874; for a non-aggregative form of lifetime view, see: Bramble 2018). Lifetime prioritarianism is typically motivated by the view that individuals persist over time as single persons, so that disadvantages at one stage of life can, in principle, be offset by advantages at another (Andrić & Herlitz 2022, p. 595).

In contrast, another cluster of views—sometimes referred to as ‘time-slice prioritarianism’—focuses on individuals’ well-being (or expected well-being) during specific periods, where a “period” may range from a moment to decades and may concern an individual’s past, present, or future. This paper concentrates on a particular variant of future-oriented version of this view.

One version common in discussions about healthcare, past-oriented prioritarianism, concentrates on past states, assuming that those who have experienced a significantly lower quality of life in the past should be treated as worse off (John et al. 2017; see also “youngest first” prioritarianism, which prioritizes those who have had the least life: Persad et al. 2009; Ferranna et al. 2022; Bognar & Hirose 2022, p. 124; cf. Bognar 2015). A second version takes into account current health states (present-oriented view) (e.g., “sickest first” prioritarianism, Persad et al. 2009; see also Segall 2016; Tännsjö 2019). The third, future-oriented view, which is the main focus of this paper in a particular form, concentrates on prospects (an ex ante version) or possible outcomes (an ex post version). The distinction between those two future-oriented options will be discussed in the next section. Of course, it is possible to distinguish other versions too (e.g., a version of time-slice future prioritarianism that cares only about the immediate future, or some specific future time span, or mixed views that include only current and future states as proposed by Nord 2005 or Herlitz 2018). Neither of these time-slice approaches has been systematically developed, as has the lifetime version of this view.

## A future-oriented chance prioritarianism

The standard version of prioritarianism can be illustrated by a hypothetical case in which, absent intervention, Alice has a lifetime well-being of 80 and Ben has 40. According to prioritarianism, an increase in lifetime well-being has greater moral value when it benefits Ben than when it benefits Alice, because the moral value of well-being gains is non-linear and depends on the recipient’s initial level. Consequently, an increase from 40 to 50 may have greater moral value than a comparable gain for Alice, such as an increase from 80 to 90. Depending on the value of the priority function, even a smaller gain for Ben may suffice to outweigh a larger gain for Alice—for example, an increase from 40 to 49 rather than from 80 to 90.

However, a different view closely related to standard prioritarianism assumes a different currency: not well-being, but chances—specifically, survival chances—that have decreasing marginal moral value. This view can be illustrated by a hypothetical case in which, without intervention, Ann’s probability of survival is 0.8 and Bob’s is 0.4, while all other morally relevant aspects of their situations are identical. On this view, an increase in survival probability generates greater moral value when it benefits Bob than when it benefits Ann. For example, increasing Bob’s survival probability from 0.4 to 0.5 will be morally more valuable than increasing Ann’s from 0.8 to 0.9. Again, depending on the value of the priority function, even a smaller gain for Bob may suffice to outweigh a larger gain for Alice—for example, an increase from 0.4 to 0.49 rather than from 0.80 to 0.90. Survival probabilities thus function as the currency to which prioritarian weighting is applied: gains in survival chances for individuals with lower baseline probabilities carry greater moral value, depending on the shape of the priority function.

It is worth noting the contrast between future-oriented chance prioritarianism and the standard version of prioritarianism, which is ex post (Adler 2012). On this view, when decisions must be made under uncertainty, the decision-maker first applies the prioritarian weighting function to the final well-being levels that individuals attain in each possible outcome. The probability of each outcome is then taken into account, and the overall evaluation is obtained by summing the probability-weighted values of these priority-weighted outcomes. Under this interpretation, the evaluation focuses on the intervention’s expected value across possible outcomes rather than on individuals’ ex ante prospects (see also Rabinowicz 2002; McCarthy 2006; Adler and Holtug 2019).

To illustrate, return to the example of Ann and Bob. Suppose that survival yields lifetime well-being of 80 and imminent death yields 40, and that, without intervention, Ann’s probability of survival is 0.8 while Bob’s is 0.4. The intervention increases the survival probability by 0.1. If the prioritarian transformation is applied to final well-being in each outcome, the expected priority-weighted value of helping Ann and helping Bob is identical, since both interventions increase the probability of the same well-being outcome by the same amount.^3^  

A different approach is ex ante prioritarianism (again as defined by Adler 2012). On this view, the prioritarian transformation is applied not to the well-being individuals ultimately realize in each outcome, but to their expected well-being. The worse off are therefore those whose ex ante prospects for well-being are lower. Priority is thus given to improving the expected well-being of individuals with the worst prospects, rather than to improving the expected value of the overall distribution of outcomes. In our example, the result is as follows: because Bob starts with lower expected well-being—his chances of reaching a well-being level of 80 are much smaller than Ann’s—improving his prospects by increasing survival probability by 0.1 receives greater moral weight under a concave prioritarian function.^4^

Future-oriented chance prioritarianism is closely related to ex ante prioritarianism, but the two views differ in the currency to which the prioritarian weighting function is applied. Ex ante prioritarianism evaluates distributions by applying the prioritarian transformation to individuals’ expected well-being, so that those with worse ex ante welfare prospects receive priority. Chance prioritarianism, by contrast, treats survival probabilities themselves as the relevant distributive currency. In many cases the two approaches will yield similar recommendations—for example, both favor helping Bob when his baseline survival probability is lower than Ann’s. However, the underlying rationale differs: ex ante prioritarianism gives priority because Bob’s expected welfare is lower, whereas chance prioritarianism gives priority because his chance of survival is lower.

Future‑oriented chance prioritarianism is therefore built on a bold premise: that survival chances themselves are genuine goods with decreasing marginal moral value. Treating chances as a distributive currency requires more than viewing them merely as instrumentally valuable predictors of outcomes, as is typically assumed in standard applications of decision theory and risk analysis. On the instrumental view, chances matter only insofar as they affect which outcomes occur; once the outcome is realized, the preceding probability distribution has no further evaluative significance. By contrast, if chances themselves are objects of distributive concern, inequalities in survival prospects must be regarded as morally significant independently of which outcomes ultimately materialize. Improvements in survival chances can therefore be beneficial not only ex ante—by increasing the likelihood of survival—but also in an ex post evaluative sense, even when the improved chances do not result in the expected outcome. Treating chances as a distributive currency thus presupposes that they are objective features of the world—analogous to physical magnitudes such as temperature—to which agents can rationally and morally attach value, rather than merely epistemic tools for predicting outcomes (see Stefánsson & Bradley 2015).

A view closely related to chance prioritarianism has been noted by several philosophers, though it has not been systematically developed. Peterson (2008), for example, introduces the idea that the moral value of increases in survival chances may be non-linear, and uses it to argue that, in some cases, a lottery that equalizes chances can be morally preferable to allocating a benefit to a single individual. This idea has been developed in more detail by Stefánsson (2024), who invokes the diminishing marginal moral value of chances to explain the so-called identifiability effect.^5^ On this account, the intuition that “we should sometimes save identified lives at the expense of saving in expectation a greater number of statistical lives” (p. 10) does not merely reflect a bias toward identifiable individuals, but rather tracks a morally relevant feature of the situation—namely, the concentration of risk. Identifiable individuals are typically those who face salient and highly concentrated risks of serious harm, whereas statistical victims represent risks that are diffuse and distributed across a population. In this way, diminishing marginal moral value of chances provides a unified explanation both for lottery-based arguments and for intuitions underlying the identifiability effect.^6^

In the next section I will illustrate some implications of future-oriented chance prioritarianism using a case related to disease prevention.

## A case study

Imagine that, at the height of a pandemic, a decision-maker must assess an individual’s risk of death from a virus *V* over some period * t* in order to allocate vaccines (ignoring other health effects). Assume that being worse off—understood in a chance-prioritarian sense—is a relevant criterion for priority setting.^7^ The risk of death depends on two factors: the probability of infection (PI) and the infection fatality rate (IFR) conditional on infection.^8^

How, then, should we determine who is worse off? While this question can be formulated at the level of individuals, public health decisions typically concern groups that differ systematically in their risk profiles—for example, younger and older cohorts. If lower survival prospects indicate being worse off, then interventions that benefit groups with lower baseline chances may deserve priority.

### Saving the younger vs. saving the older

To illustrate this issue, consider two age cohorts and the question of which should be prioritized for vaccination when resources are limited: 55–64 and 65–74. Their estimated infection fatality rates (IFRs) are 0.75% and 2.5% respectively (based on Levin et al. 2020). One might think that every individual in the older group is automatically worse off, since their IFR is higher. However, what also matters is the probability of infection (PI). If the younger group is more exposed—for example, because many are still in the workforce—then their overall risk of death from the virus can be higher.

Suppose PI is 0.334 for the younger cohort and 0.10 for the older. Table 1 summarizes the results. Although the older group has a higher IFR, the higher exposure of the younger group means that they face more expected deaths overall: 2,505 compared with 2,500. In other words, vaccinating the younger cohort could save more lives, despite their lower IFR.

Now assume that a vaccine is available that is equally effective for both groups (for simplicity, 100% effective). If only one cohort can be prioritized, who should it be? Several reasons might favor the younger cohort:

- 1A. More lives saved: 2,505 vs. 2,500 deaths prevented.
- 1B. More life-years saved: younger people, on average, would live longer if protected.
- 1C. More QALYs saved: younger survivors are expected to have more years in good health.
- 1D. Higher individual risk: every member of the younger cohort has a slightly greater chance of dying (0.002505 vs. 0.0025).
- 1E. More deaths prevented per 1,000 people: 2.505 vs. 2.5.

At first glance, the first three reasons (1 A–1 C) are utilitarian, as they aim to maximize aggregate health benefits (lives, life-years, or QALYs). By contrast, 1D and 1E highlight a different evaluative dimension—individual or group risk. While 1D focuses on each person’s probability of death, 1E concerns expected deaths per fixed population. Although these rely on different interpretations of probability, in this case they support the same conclusion: prioritizing the younger cohort.

This section has evaluated prospects of death using whole cohorts as the relevant units of comparison. The next section considers an alternative approach that focuses instead on those who are actually exposed to the risk.

**Table 1:** Estimated risks of death from virus V in two cohorts (if unvaccinated)

		1. Cohort 55–64	2. Cohort 65–74
1.	Population size	1,000,000	1,000,000
2.	Probability of infection (PI)	0.334	0.1
3.	Infection fatality rate (IFR)	0.75%	2.5%
4.	Expected infections	334,000	100,000
5.	Expected deaths	2,505	2,500
6.	Individual probability of death*	0.002505	0.0025

*Probability of dying from V within period t if not vaccinated.

### Delineating the worse off and subpopulation choice

Instead of comparing whole cohorts, we might restrict attention to those individuals who are actually infected within period *t*, and treat this subpopulation as the relevant group of worse-off individuals.^9^ If we apply this approach, infected individuals belonging to the older cohort seem to be worse off in the relevant sense than infected individuals belonging to the younger cohort. Among infected individuals, those from the older cohort face a higher probability of death (2.5% vs. 0.75%) than those from the younger cohort. In this scenario there are at least two reasons that favor the older cohort:

- 2D. Greater individual risk reduction: vaccinating infected members of the older cohort reduces a higher probability of death from being infected (0.025 vs. 0.0075).
- 2E. More deaths prevented among the infected per 1,000 infected individuals: vaccination would save 2.5 lives in the older cohort but only on average 0.75 in the younger.

These recommendations diverge from utilitarian approaches. While anyone who dies from the virus must belong to the infected subpopulation, utilitarian evaluation proceeds at the level of the whole population, comparing aggregate outcomes such as total deaths avoided, lives saved, or QALYs saved. By contrast, chance-prioritarian reasoning does not aim to maximize aggregate outcomes, but instead assesses priority by comparing how badly off individuals are with respect to survival prospects, here understood conditionally on infection.

This example also illustrates a crucial and often overlooked feature of priority setting in preventive contexts: before applying any distributive principle, one must determine how to delineate the relevant group of worse-off individuals. In our case, one can focus on entire cohorts (as in Sect. 3.1) or only on those who will in fact become infected (as here). This choice reflects substantive judgments about which features of the situation are morally relevant. Importantly, these judgments are not determined by prioritarianism itself, but by background assumptions about how the relevant population is defined.

## Discussion

This section builds on the preceding case study to examine the conceptual foundations of chance prioritarianism. The case study reveals a central tension: policies that reduce the total number of deaths (or related aggregate health outcomes) may do so either by targeting individuals with higher overall risk or by addressing more severe risks within particular subpopulations, depending on how risk is defined and how the population is partitioned. This motivates two main questions. (1) Risk concentration: Why, exactly, does exposure to a higher risk (e.g., of death) make an individual worse-off, and what is the appropriate normative weight of such risk concentration in our evaluations? (2) Partitioning the population: Should distributive comparisons in the above hypothetical example track entire cohorts or only the infected subpopulation, and on what grounds? Addressing these points inevitably raises issues about the interpretation of probability: in what sense do individuals have higher or lower chances of death, as these chances—understood as objective, subjective, or evidential—may serve as the currency (informational basis) of distributive justice?

### Chance prioritarianism and the normative strength of risk concentration

For a decision-maker, the practical question is which cohort—younger or older—should be prioritized, and on what grounds. Allowing the concentration of serious risks on a small number of individuals in order to maximize aggregate benefits may be morally objectionable, and distributing risks more evenly may therefore be preferable. But what is the normative weight of risk concentration, particularly when distributing risks more evenly leads to fewer lives being saved?

By analogy with standard prioritarianism that concerns welfare, future-oriented chance prioritarianism allows departures from saving the greatest number when an act benefits those with worse survival prospects. Views on the normative significance of risk concentration range widely. At one end, some authors treat it merely as a tie-breaker. Daniels (2012), for example, suggests that “risk concentration might be such a weak factor that it matters morally only in breaking ties” (p. 41), which implies that when the choice is between saving more lives and aiding those with worse prospects, we should still maximize lives saved. Lazar (2018) makes a similar point, entertaining the idea that concentrated risks might warrant some priority, perhaps on fairness grounds, but ultimately concluding that their moral significance is “no more than a tie-breaker” (p. 153).

At the other end of the spectrum, John (2014), Frick (2015), Otsuka (2015), and Stefánsson (2024), argue that risk concentration can carry decisive moral weight, albeit for different reasons. John (2014), for instance, appeals to Rose’s “prevention paradox” (Rose, Khaw et al. 2008) and a contractualist framework to defend the principle that “we should help those at greater risk of serious harm rather than those at lower risk of equivalent harm” (p. 29). Frick (2015) advances a similar claim from an ex ante contractualist perspective, emphasizing that what matters for justification is the fairness of prospects rather than merely outcomes. Contractualists generally hold that moral principles must be justifiable to each person. From this perspective, policies that concentrate serious risks on some individuals are harder to justify than those that distribute risk more evenly—even when the latter result in greater total harm ex post. The reason is that the ex ante complaint of a person left with a high risk is stronger than the complaint of someone bearing a modest increase in a small risk. The difficulty, however, of this view is that it does not provide a principled method for determining how to weigh such complaints against “well-being-given reasons” that favor saving more lives (Frick 2015, p. 196).

A benefit of explaining the normative significance of risk concentration by the decreasing marginal moral value of survival chances—rather than by contractualist reasons referring to individual claims—is its precision. Suppose we take a concave priority function based on the square root, *u(s) = √s* (where *s* is survival probability). Conditional on infection, the stakes are higher for the older cohort (see Table 1): a fully effective vaccine would prevent about 250 deaths per 10,000 infected older individuals, compared with 75 among the younger. Given the number of infections, this corresponds to roughly 2,500 deaths averted among older individuals and 2,505 among younger individuals.

Now apply the square-root valuation. The per-infected moral gain is about 0.01258 for an older person (since 1 − √0.975) and about 0.00376 for a younger person (since 1 − √0.9925). Aggregating over the infected yields total prioritarian gains of about 1,257.91 (older) versus 1,254.86 (younger)—slightly favoring the older cohort.

By contrast, when we treat the whole cohorts as the relevant populations (1,000,000 each), the per-person gains are about 0.0012508 for the older cohort (since 1 − √0.9975) and about 0.0012533 for the younger cohort (since 1 − √0.997495). This produces aggregated values of about 1,250.78 (older) versus 1,253.29 (younger), now favoring the younger cohort.

Under the same concave risk-weighting function, the infected-subpopulation comparison gives greater weight to the older cohort, whereas the whole-cohort comparison supports prioritizing the younger. This shows how the choice of reference class—infected versus whole cohort—can interact with the priority function to generate divergent distributive recommendations. This leads us to the problem of partitioning the population.

### Partitioning the population: causal sensitivity and interpretations of probability

More generally, chance prioritarianism’s precision about the normative significance of risk concentration coexists with a dependence on how the population is partitioned: alternative, prima facie reasonable divisions—conditioning on infection rather than exposure, using whole-population rather than case-only baselines, grouping by age bands or by comorbidity strata, or evaluating ex ante rather than post-allocation—apply the same concave weights to different denominators and baselines and can therefore deliver different verdicts. In extreme cases this produces Simpson’s-paradox–style reversals: a cohort that is priority-worthy within the infected subpopulation may cease to be so when assessed over the whole cohort, and vice versa. The view is therefore partition-sensitive: fixing the priority function does not, by itself, fix the ranking of allocations. I do not resolve this dependence here; my aim is to register the methodological upshot and to motivate the subsequent question of which delineation is more appropriate.

One may argue that it seems reasonable to assume that actually being infected with V—rather than merely belonging to an age cohort—renders an individual worse off in the relevant sense, namely with respect to survival chances. Infection is the step on the causal pathway to V-related death: conditional on not being infected, the probability of V-related death is (for practical purposes) 0, whereas conditional on being infected it equals the infection fatality rate (IFR). This consideration therefore supports the claim that a decision-maker should direct moral attention to those who are (or will be) infected, even if their identities are presently unknown—or indeed unknowable ex ante—given the unpredictability of social contacts and patterns of spread. The rationale is simple: individuals who will not be infected with V bear no conditional risk of death from the virus. Being infected is therefore what renders an individual worse off in the relevant sense, since infection—rather than mere membership in an age cohort—is what causally leads to death in some cases.

This approach seems to align with the propensity interpretation of probability (expressed by 2D), in which probabilities are understood as tendencies or dispositions of individuals. Saying that a patient has a 2.5% probability of death if infected means this individual has a biological propensity—grounded in physiology—that would manifest as death in about 1 out of 40 comparable infection episodes. In other words, an analogy with a casino makes the idea vivid: a large roulette wheel divided into 1,000 slots, only 25 of which are red, has a 2.5% propensity to stop on red.

This interpretation also makes sense of ordinary clinical language. Physicians often say to their patients that “smoking significantly increases your risk of lung cancer” or that “lowering cholesterol reduces your risk of heart attack.” These statements—setting aside, for the moment, the evidence supporting them—are best understood not as claims about group-level frequencies, but as accounts of how an intervention alters an individual’s underlying biological disposition to develop disease. Similarly, when a clinician told a patient in 2021 that “vaccination lowers your risk of severe COVID-19,” (in our hypothetical example it lowers to 0) the most straightforward reading is that vaccination could reduce that person’s propensity to experience the pathological processes. In severe cases, death could result from lymphocyte-containing inflammatory infiltrates in the lung, a cytokine storm, and the accumulation of hyaluronic acid that contributes to respiratory failure.

From this perspective, one may argue that prioritizing the younger cohort incorrectly extrapolates risk to every member of the two cohorts. It is not morally relevant that everyone in the younger cohort has an unconditional probability of V-related death equal to 0.002505, while everyone in the older cohort has slightly lower: 0.0025 (see Table 1, line 6). What is morally relevant is that each infected individual in the younger cohort (i.e., 334,000 people) faces a much lower infection fatality rate than those infected in the older cohort (i.e., 100,000 people): 0.0075 versus 0.025 (see Table 1, line 3). In other words, what matters for assessing risk concentration is not line 5 (total expected deaths) or line 6 (each cohort member’s unconditional probability of death), but line 3 (the IFR among the infected). This way of framing the problem supports Option 2D presented in Sect. 3, which prioritizes those who, once infected, face the greater individual risk of death. This shift of focus from unconditional probabilities to conditional infection fatality rates highlights how different delineations of the worse off lead to different distributive recommendations.

However, a main problem with this interpretation is that biomedical mechanisms need not be genuinely “chancy” in the metaphysical sense. Outcomes like cytokine storms or cholesterol-driven heart attacks may be better understood as deterministic causal processes rather than lotteries (exemplified above with a large roulette wheel). Moreover, even if physicians’ language in clinical practice may suggest propensities as the interpretation of probability claims, such propensities are not directly known to physicians (at least in our example of a viral infection). They are typically not measurable in principle, and clinical or epidemiological practice therefore infers them indirectly from population-level data rather than observing them at the individual level. Therefore, contemporary evidence-based medicine does not require mechanistic knowledge in order to make probabilistic predictions in the case of individual patients: it relies on epidemiological evidence that measures group-level frequencies derived from clinical trial data, rather than on detailed mechanistic accounts of why, for example, a cytokine storm develops more likely in one patient but not in another.

This frequentist view better aligns with the methodology of evidence-based medicine, as regulatory frameworks adopted by the U.S. Food and Drug Administration (FDA) and the European Medicines Agency (EMA) rely on population-level statistical inference rather than on the direct measurement of individual causal propensities. Clinical trials produce measures such as absolute risk (AR) and absolute risk reduction (ARR), which are then generalized to individual patients (more on this, see Fuller 2020). For example, vaccination effectiveness is established in population studies, and vaccination guidelines then ascribe a corresponding “risk” to individual patients and assess how much a vaccine reduces that risk. Saying that the IFR for a given cohort is 2.5% means simply that, in a large enough population, 2.5% of those infected will die, not that each individual has 2.5% propensity to die. Probabilities are not properties of individuals but of cohorts or sequences of cases. Therefore, it may seem that the more suitable justification of chance-prioritarian choice would be 2E that refers to the expected number of deaths per fixed group of people.

However, this also poses a problem. On a chance-prioritarian view we are interested in the features that make particular individuals worse off. The relevant object seems to be single-case probability, not merely a rate relative to some reference class, whereas 2E grounds priority in group frequencies. In our example, a reference to 2E appeals to the greater deaths averted among infected older people (≈ 2.5 per 1,000) than among infected younger people (≈ 0.75 per 1,000). But that rationale no longer turns on what marks an individual as worse off—being infected—which originally motivated 2D; rather, it trades on the performance of a group, raising the question of why this does not collapse into some option 1 rationale (“lives saved”). On 2E we risk losing the person-affecting basis: individuals benefit not because of their own higher conditional risk but because of the cohort to which they belong.

The chance prioritarianism thus faces a dilemma. If the currency is each person’s survival chance, D-type justifications fit best, and—under weak assumptions about the meaning of priority function—2D seems most apt, since only the infected are genuinely at risk of V-death. Yet current medical practice does not furnish individual-level propensities (at least in the discussed types of illness); decision-makers typically have cohort-based estimates (as in Sect. 3). Hence the case for D, and especially 2D, leans on idealized information that current evidential practice in healthcare does not provide.

However, frequentism that would justify options E also faces well-known difficulties, stemming from the fact that any individual belongs to multiple reference classes—by age, sex, diet, genetics, occupation, and so on—and risk predictions vary with the class chosen (see Hájek 2007 for an argument that the reference class problem is much broader than it may seem and concerns many other interpretations of probability). Let as assume that we vaccinate the older cohort. For a statistical 70-year-old member of this cohort, their unconditional probability of death falls from 0.0025 to 0, yielding a modest gain. If instead we treat them as part of the infected subpopulation of this cohort, their conditional probability of death drops from 0.025 to 0, a ten-fold larger gain. Both descriptions are “true”, but they imply very different distributive priorities.

It is surprising that the reference class problem, a problem rather well-known to epistemologists, rears its head with little notice in discussed contexts. When a problem is modeled with only a single variable (e.g. age cohort), questions about group membership—or “reference class”—don’t arise, and calibration within that variable seems to indicate what’s rational to believe about an individual. However, this artificially narrow setup masks a deeper issue: in real contexts, individuals belong to multiple intersecting groups (in our highly stylized example: two age cohorts and infected or no infected). The reference class problem becomes unavoidable once this complexity is acknowledged, because it is unclear which group or groups should determine the basis for calibration. Thus, knowing that a model is calibrated by age and infection alone doesn’t tell you what it implies about an individual who falls into many categories. The problem is thus not statistical but normative: which reference class is relevant for adjusting an individual’s score? The central difficulty is that probabilistic reasoning in ethics is unavoidably mediated by background judgments about which group memberships are normatively salient.^10^

## Conclusion

This paper has argued that a number of real-world prioritization patterns in preventive healthcare—most notably those that give priority to individuals with lower survival prospects—can be understood as reflecting a form of future-oriented chance prioritarianism. On this view, survival probabilities function as the relevant distributive currency, and improvements in these probabilities carry greater moral weight when they benefit those whose baseline chances are lower.

At the same time, the analysis has shown that applying this view is not straightforward. The central difficulty is not simply how much weight should be given to risk concentration, but how to identify the relevant bearers of risk in the first place. That issue cannot be separated from the interpretation of probability. Whether survival chances are understood as properties of individuals or as frequencies defined over groups shapes which population partition becomes normatively salient—for example, whether the relevant comparison is between whole cohorts or between infected subpopulations. Different interpretations of probability therefore support different ways of delineating who counts as worse off, and can thereby generate conflicting distributive recommendations even under the same priority function.

Taken together, these points suggest that chance prioritarianism provides a useful lens for interpreting priority-setting in preventive contexts, but that its application is not straightforward. Its recommendations depend not only on the shape of the priority function, but also on prior judgments about how risks are distributed, how populations are partitioned, and how probabilities are to be understood.

## Footnotes

1 Arras, 2005, p. 285; Bunnik, 2023, p. 193; Curran & John, 2022, p. 689; Verweij, 2009, p. 164.

2 Chance prioritarianism may also refer to other goods than survival, see: Macklin & Cowan, 2012, p. 1542.

3 Let *x*_*A*_ be the option of helping Ann and *x*_*B*_ the option of helping Bob, and let g(⋅) be the prioritarian transformation applied to the components of each well-being vector *w(x)*. If survival yields lifetime well-being 80 and death 40, then under *x*_*A*_ the expected sum of priority-weighted well-being numbers is: 0.9 g(80) + 0.1 g(40) + 0.4 g(80) + 0.6 g(40) = 1.3 g(80) + 0.7 g(40). Whereas under $\:{x}_{B}$ it is: 0.8 g(80) + 0.2 g(40) + 0.5 g(80) + 0.5 g(40) = 1.3 g(80) + 0.7 g(40). The two options therefore receive the same ex post prioritarian evaluation.

4 Their expected well-being without intervention is therefore 0.8 × 80 + 0.2 × 40 = 72 for Ann and 0.4 × 80 + 0.6 × 40 = 56 for Bob. Bob is thus worse off in ex ante terms, which explains why improving his prospects receives greater moral weight under a concave prioritarian function.

5 For a critical review of different arguments in favour of a preference for identified victims, see Żuradzki, 2019.

6 A related idea—that chances may have decreasing marginal value—has also been developed in decision theory, where it is used to explain rational aversion to risk and ambiguity, e.g., the Ellsberg and Allais paradoxes (Bradley, 2016; Nissan-Rozen, 2019; Bradley & Stefánsson, 2023; Stefánsson, 2023).

7 Of course, in real decisions this is only one of many other criteria; for example, Persad et al., (2009) in their well-known paper enumerate three others: treating people equally, maximizing total benefits, and promoting and rewarding social usefulness.

8 In practice, estimates of IFR are often more reliable (based on large meta-analyses) than estimates of PI, which may rely on limited or uncertain data.

9 This idea is inspired by Rose’s prevention paradox (Rose et al., 2008), which highlights that most cases of disease may arise from populations at low individual risk. Whereas Rose’s framework motivates population-wide interventions aimed at minimizing aggregate disease burden, chance prioritarian approach treats high individual risk—conditional on exposure—as morally salient and grounds priority in the severity of survival prospects rather than in overall case reduction.

10 For a case study illustrating how such classifications can shape biomedical research and healthcare decisions, see Żuradzki & Malinowska, 2024.

## References

[CR1] Adler, M. 2012. *Well-Being and Fair Distribution*. Oxford: Oxford University Press.

[CR2] Adler, M. 2019. *Measuring Social Welfare: An Introduction*. Oxford: Oxford University Press.

[CR4] Adler, M., and N. Holtug. 2019. Prioritarianism: A response to critics. *Politics, Philosophy & Economics* 18 (2): 101–144.

[CR3] Adler M.D. (2022). Theory of prioritarianism. In: Matthew D. Adler and Ole F. Norheim, eds., *Prioritarianism in practice* (pp. 37–127), Cambridge University Press.

[CR5] Andrić, V., and A. Herlitz. 2022. Prioritarianism, time-slices, and prudential value. *Australasian Journal of Philosophy* 100 (3): 595–604.

[CR6] Arras, J.D. 2005. Rationing vaccine during an avian influenza pandemic: Why it won’t be easy. *The Yale Journal of Biology and Medicine* 78 (5): 287. PMC2259161 17132335

[CR7] Bognar, G. 2015. Fair innings. *Bioethics* 29 (4): 251–61. 10.1111/bioe.12101 24909449

[CR8] Bognar, G., and I. Hirose. 2022. *The ethics of health care rationing: an introduction*. Routledge.

[CR9] Bradley, R. 2016. Ellsberg’s paradox and the value of chances. *Economics & Philosophy* 32 (2): 231–248.

[CR10] Bradley, R., and H.O. Stefánsson. 2023. Fairness and risk attitudes. *Philosophical Studies* 180 (10): 3179–3204.

[CR11] Bramble, B. 2018. *The passing of temporal well-being*. Routledge.

[CR13] Bubar, K.M., K. Reinholt, S.M. Kissler, et al. 2021. Model-informed COVID-19 vaccine prioritization strategies by age and serostatus. *Science* 371 (6352): 916–921. 10.1126/science.abe6959 PMC7963218 33479118

[CR14] Bunnik, E.M. 2023. Ethics of allocation of donor organs. *Current Opinion in Organ Transplantation* 28 (3): 192. 10.1097/MOT.0000000000001058 PMC10155689 36787240

[CR15] Curran, E.J., and S.D. John. 2022. Must we vaccinate the most vulnerable? Efficiency, Priority, and Equality in the Distribution of Vaccines. *Journal of Applied Philosophy* 39: 682–697. 10.1111/japp.12588 PMC9347581 35937030

[CR16] Daniels, N. 2012. Reasonable disagreement about identified vs. statistical victims. *Hastings Center Report* 42 (1): 35–45. 10.1002/hast.13 22616399

[CR17] Faust, H.S., & Menzel, P.T. (Eds.). (2011). *Prevention vs. treatment: What’s the right balance?*. Oxford University Press.

[CR18] Ferranna, M., J.P. Sevilla, and D.E. Bloom. 2022. Addressing the COVID-19 pandemic: Comparing alternative value frameworks. In *Prioritarianism in practice*, ed. M.D. Adler and O.F. Norheim, 360–407. Cambridge: Cambridge University Press.

[CR19] Frick, J. 2015. Contractualism and social risk. *Philosophy and Public Affairs* 43: 175–223.

[CR20] Fuller, J. 2020. Epidemiological evidence: Use at your ‘own risk’? *Philosophy of Science* 87 (5): 1119–1129.

[CR21] Galvani, A.P., T.C. Reluga, and G.B. Chapman. 2007. Long-standing influenza vaccination policy is in accord with individual self-interest but not with the utilitarian optimum. *Proceedings of the National Academy of Sciences* 104 (13): 5692–5697. 10.1073/pnas.0606774104 PMC1838447 17369367

[CR22] Giubilini, A., J. Savulescu, and D. Wilkinson. 2021. Queue questions: Ethics of COVID-19 vaccine prioritization. *Bioethics* 35 (4): 348–55. 10.1111/bioe.12858 PMC8013927 33559129

[CR24] Herlitz, A. 2018. Health, priority to the worse off, and time. *Medicine, Health Care and Philosophy* 21 (4): 517–27. 10.1007/s11019-018-9825-2 PMC6267230 29350341

[CR23] Hájek, A. 2007. The reference class problem is your problem too. *Synthese* 156 (3): 563–585.

[CR59] Holtug, N. 2003. Welfarism – The very idea. *Utilitas* 15 (2): 151–174.

[CR27] John, S.D. 2014. Risk, contractualism, and Rose’s ‘Prevention Paradox.’ *Social Theory and Practice* 40 (1): 28–50.

[CR28] John, S.D. (2025). Prevention, treatment, and cost. In: A. Broadband (Ed.), *The Oxford handbook of philosophy of medicine* (pp. 263–280), Oxford University Press.

[CR29] John, T.M., J. Millum, and D. Wasserman. 2017. How to allocate scarce health resources without discriminating against people with disabilities. *Economics and Philosophy* 33 (2): 161–186.

[CR30] Lazar, S. 2018. Limited aggregation and risk. *Philosophy & Public Affairs* 46 (2): 117–159.

[CR31] Levin, A., W.P. Hanage, N. Owusu-Boaitey, et al. 2020. Assessing the age specificity of infection fatality rates for COVID-19: Systematic review, meta-analysis, and public policy implications. *European Journal of Epidemiology* 35: 1123–1138. 10.1007/s10654-020-00698-1 PMC7721859 33289900

[CR32] Macklin, R., and E. Cowan. 2012. Given financial constraints, it would be unethical to divert antiretroviral drugs from treatment to prevention. *Health Affairs* 31 (7): 1537–1544. 10.1377/hlthaff.2012.0071 PMC3572856 22778343

[CR33] McCarthy, D. 2006. Utilitarianism and prioritarianism. *Economics and Philosophy* 22 (3): 335–363.

[CR34] McKerlie, D. 1997. Priority and time. *Canadian Journal of Philosophy* 27 (3): 287–309.

[CR35] Nielsen, L. 2022. Pandemic prioritarianism. *Journal of Medical Ethics* 48: 236–239. 10.1136/medethics-2020-106910 33542106

[CR36] Nissan-Rozen, I. 2019. The value of chance and the satisfaction of claims. *Journal of Philosophy* 116 (9): 469–493.

[CR37] Nord, E. 2005. Concerns for the worse off: Fair innings versus severity. *Social Science & Medicine* 60 (2): 257–63. 10.1016/j.socscimed.2004.05.003 15522483

[CR38] Otsuka, M. (2015). Risking life and limb: How to discount harms by their improbability. In I. Glenn Cohen, Norman Daniels, & Nir Eyal (Eds.), *Identified versus statistical lives* (pp. 77–93). Oxford University Press.

[CR40] Ottersen, T. 2013. Lifetime QALY prioritarianism in priority setting. *Journal of Medical Ethics* 39 (3): 175–80. 10.1136/medethics-2012-100740 23204320

[CR41] Parfit, D. 1997. Equality and priority. *Ratio* 3: 202–21.

[CR42] Parfit, D. 2012. Another defence of the priority view. *Utilitas* 24 (3): 399–440.

[CR43] Persad, G., A. Wertheimer, and E.J. Emanuel. 2009. Principles for allocation of scarce medical interventions. *Lancet* 373 (9661): 423–31. 10.1016/S0140-6736(09)60137-9 19186274

[CR44] Peterson, M. 2008. “The moral importance of selecting people randomly.” *Bioethics* 22 (6): 321–327. 10.1111/j.1467-8519.2008.00636.x 18445094

[CR45] Ponthiere, G. (2016). Lifetime well-being. In: *The Oxford handbook of well-being and public policy*. M.D. Adler and M. Fleurbaey. Oxford, Oxford University Press.

[CR46] Rabinowicz, W. 2002. Prioritarianism for prospects. *Utilitas* 14 (1): 2–21.

[CR47] Rose, G.A., K.-T. Khaw, and M. Marmot. 2008. *Rose’s strategy of preventive medicine*. Oxford University Press.

[CR48] Segall, S. 2016. *Why inequality matters*. Cambridge University Press.

[CR49] Sharp, D., and J. Millum. 2018. Prioritarianism for global health investments: Identifying the worst off. *Journal of Applied Philosophy* 35 (1): 112–132.

[CR50] Smith, N.M., J.S. Bresee, D.K. Shay, T.M. Uyeki, N.J. Cox, and R.A. Strikas. 2006. Prevention and control of influenza: Recommendations of the advisory committee on immunization practices (ACIP). *MMWR Recommendations and Reports* 55 ((RR-10)): 1–42. 16874296

[CR51] Stefánsson, H.O. 2023. In defence of Pigou-Dalton for chances. *Utilitas* 35 (4): 292–311.

[CR52] Stefánsson, H.O. 2024. Identified person “bias” as decreasing marginal value of chances. *Noûs* 58 (2): 536–561.

[CR53] Stefánsson, H.O., and R. Bradley. 2015. How valuable are chances? *Philosophy of Science* 82 (4): 602–625.

[CR54] Tännsjö, T. 2019. *Setting healthcare priorities: What ethical theories tell us*. Oxford University Press.

[CR55] Verweij, M. 2009. Moral principles for allocating scarce medical resources in an influenza pandemic. *Journal of Bioethical Inquiry* 6: 159–169.

[CR56] Wiśniowska, K., T. Żuradzki, and W. Ciszewski. 2022. Value choices in European COVID-19 vaccination schedules: How vaccination prioritization differs from other forms of priority setting. *Journal of Law and the Biosciences* 9 (2): lsac026. 10.1093/jlb/lsac026 PMC9512243 36172170

[CR57] Żuradzki, T. 2019. The normative significance of identifiability. *Ethics and Information Technology* 21 (4): 295–305.

[CR58] Żuradzki, T., and J.K. Malinowska. 2024. Ethno-racial categorisations for biomedical studies: The fair selection of research participants and population stratification. *Synthese* 204: 130. 10.1007/s11229-024-04769-8 PMC11447102 39372679

